# Epidemiology Tools to Evaluate the Control of Proliferative Enteropathy in Commercial Pig Herds

**DOI:** 10.3390/ani14091357

**Published:** 2024-04-30

**Authors:** Alison Collins, Cherie Collins

**Affiliations:** 1 New South Wales Department of Primary Industries, Elizabeth Macarthur Agricultural Institute, Menangle, NSW 2568, Australia; 2 Rivalea Australia, P.O. Box 78, Corowa, NSW 2646, Australia; ccollins@rivalea.com.au

**Keywords:** proliferative enteropathy (PE), *Lawsonia intracellularis*, disinfection, vaccination, seroconversion, diarrhea, hemorrhage, olaquindox, epidemiology

## Abstract

**Simple Summary:**

Evaluating disease control in commercial pig herds requires the use of infection surveillance tools as well as observing clinical signs and measuring production changes. Many disease control studies evaluate treatments in research facilities where conditions do not equate with commercial farms, specifically with respect to the presence of multiple and repeated challenges to pigs. This study evaluated the control of the wasting disease proliferative enteropathy with disinfection, vaccination, and/or medication in 84 pig pens under commercial conditions using epidemiological tools. Vaccinated pigs housed in lime-disinfected pens showed the best control of proliferative enteropathy with reduced diarrhea and fecal shedding of *Lawsonia intracellularis* and an increased growth rate and prevalence of protective antibodies. Continuous medication with olaquindox prevented *L. intracellularis* infection until it was removed at 17 weeks of age, leaving naïve finisher pigs susceptible to intestinal hemorrhage, bloody feces, reduced growth, and mortalities. To avoid proliferative enteropathy, control strategies need to suppress but not prevent *L. intracellularis* infection, while enabling protective antibodies to develop. This study demonstrated the value of monitoring the timing and level of excretion of *L. intracellularis* in pig pens to compare proliferative enteropathy control on commercial farms.

**Abstract:**

Proliferative enteropathy (PE) is characterized by diarrhea and reduced weight gains in growing pigs and intestinal hemorrhage in finishers. Vaccination, antibiotic medication, and improved hygiene can control PE, but their efficacy depends upon the epidemiology of PE. This study monitored the timing and severity of PE in 84 commercial pens across seven treatments, including disinfection, vaccination, no treatment, medication with olaquindox (50, 25 and 12.5 ppm), and combined disinfection and vaccination. Vaccination with or without lime disinfection suppressed clinical signs of PE and reduced the number of excreted *L. intracellularis* relative to untreated pigs housed in cleaned or cleaned and disinfected pens between 9 and 17 weeks of age. Continuous olaquindox mediation to 17 weeks of age prevented *L. intracellularis* infection, leaving finisher pigs naïve. These finisher pigs suffered an outbreak of hemorrhagic enteropathy with significant reductions in weight gain, feed intake, and mortalities of 4.6%. Over the 13 week grow/finish period, vaccinated pigs housed in disinfected pens showed significantly higher weight gain and feed intake relative to all other treatments, equating to a weight gain difference of between 3.6 and 3.9 kg per pig. Monitoring the immune response and fecal excretion of *L. intracellularis* in pens of pigs enabled effective PE control strategies to be evaluated on the farm.

## 1. Introduction

Proliferative enteropathy (PE) is a wasting disease of grower and finisher pigs caused by *Lawsonia intracellularis*, an intracellular bacterium that replicates in the cytoplasm of epithelial cells lining the ileum and causes thickening of the mucosa due to proliferation of immature enterocytes [1,2]. The clinical signs of PE depend on the dose of *L. intracellularis* [3,4,5] and the age and susceptibility of pigs, ranging from diarrhea and reduced growth rates in grower pigs to acute hemorrhagic enteropathy and mortality in up to 10% of affected finisher pigs [6,7]. Economic losses associated with clinical and sub-clinical PE have been estimated at between AUD 8 and AUD 13 per pig (between USD 5 and USD 8.30) in reduced net revenue [8]. Losses are due to reduced weight gains, variation in pig weights within batches, poor feed efficiency, mortalities, and increased days to slaughter.

Serological surveys have demonstrated between 80% and 100% of pig herds are infected with *L. intracellularis* [9,10] with an average of 84% of finisher pigs infected within herds. Pigs are susceptible to *L. intracellularis* after weaning, with the loss of maternal antibodies provided in dams’ milk [11]. However, the common inclusion of antibiotics in weaner diets to control other enteric or respiratory diseases may prevent *L. intracellularis* infection until pigs are moved to grower or finisher accommodation. Serological surveys on commercial pig farms demonstrate an increased seroprevalence to *L. intracellularis* in older animals [10,12,13,14,15]. *L. intracellularis* is transmitted between pigs by the fecal-oral route [4,16,17], so poor pen hygiene, continuous flow production, an accumulation of manure, and the mixing of pigs increases the risk of *L. intracellularis* infection [13,18,19]. Natural exposure to *L. intracellularis* and vaccination induce protective immunity to *L. intracellularis*, including specific antibodies (IgA and IgG) and cell-mediated (interferon gamma) immune responses in serum and intestinal mucosa [4,20,21,22,23].

Experimental infection studies have demonstrated that vaccination [20] and antibiotic medication [24,25,26,27,28,29] can reduce clinical signs of PE, reduce fecal excretion of *L. intracellularis* and reduce histopathologic lesions of PE in affected pigs. However, many PE control studies evaluate treatments in research facilities where conditions do not equate with commercial farms, specifically with respect to the presence of multiple and repeated challenges to pigs, stocking density, hygiene, and environmental conditions. In addition, experimental challenge studies that define PE control as the reduced severity of histopathology lesions require necropsy of pigs at the peak of intestinal pathology and may thus underestimate the production losses associated with a more protracted recovery. 

This study aimed to demonstrate the value of molecular and serological epidemiological tools to compare the efficacy of PE control strategies commonly used on commercial pig farms, noting that fecal excretion of *L. intracellularis* has been proven to be correlated with the severity of PE lesions and clinical signs of PE [30,31,32,33]. PE control strategies to be evaluated include the published treatments of olaquindox [29,34], vaccination with Enterisol^®^ Ileitis [20], disinfection [35], and tylosin [25]. 

## 2. Materials and Methods

### 2.1. Animals, Housing, and Experimental Design

The study was conducted according to the Australian Code for the Care and Use of Animals for Scientific Purposes, and animal ethics approval was granted by the Animal Ethics Committees of both the commercial pig production company and the Elizabeth Macarthur Agricultural Institute (13V057C). Approximately 3024 grower pigs (9-week-old females and males) were housed in 84 pens (42 male and 42 female) in a high health status commercial piggery in south-eastern Australia. Each pen was allocated to one of seven treatments (Table 1). All pens were pressure washed with water prior to filling over two weeks with three replicates each week, equaling a total of six replicates. Pens had a mix of solid and concrete slatted floors (33:66) with mesh dividers between each pen. Approximately 35 ± 2 pigs were housed in each pen and pigs had ad libitum access to feed and water. The base grower diet was comprised mainly of wheat, canola meal, meat meal, and tallow and was formulated to meet the animals’ requirements, including 10.1 MJ net energy per kg, 17.7% crude protein, 2.97% fat, 48.35% starch, 3.8% fiber, and 1.12% lysine. Finisher base diets were primarily composed of wheat, barley, canola meal, meat meal, and tallow and were formulated to 9.94 MJ net energy per kg, 14.2% crude protein, 1.96% fat, 51.55% starch, 3.75% fiber, and 0.87% lysine. 

**Figure 1:**
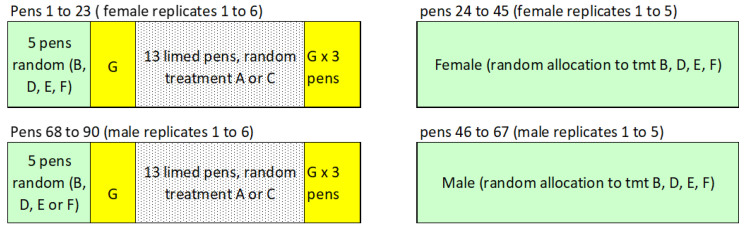
Schematic diagram of pens and treatment layout within sheds.

All pigs were medicated with 50 ppm olaquindox (Keyquindox, International Animal Health Products, Huntingwood, NSW, Australia) from 3 to 9 weeks of age, prior to the start of the trial. Pigs in treatment groups A and B were vaccinated orally with Enterisol^®^ Ileitis (Boehringer Ingelheim Animal Health USA Inc., Duluth, GA, USA) between two and three weeks of age, leaving a three-day antibiotic-free window before and after vaccination. Pens for treatments A and C were disinfected with lime (Hydrated lime, plasterer’s quality) prior to the grower pigs entering at 9 weeks of age. These lime-disinfected pens were grouped together to avoid cross-contamination with other pens. The pens allocated to 50 ppm of Olaquindox were also grouped together and placed adjacent to the lime-treated pens with solid dividers between treatments. All other treatments were randomly allocated to the remaining pens (Figure 1).

Olaquindox was maintained in the diets of treatment E, F, and G pigs until day 56 of the trial (16–17 weeks of age) when it was replaced with a two-day pulse of 400 ppm tylosin (Tylan 250, Elanco @ 16 mg/kg bodyweight, Greenfield, IN, USA) in feed every 10 days from trial day 56 until sale at 21–22 weeks of age. Tylan was also pulsed in feed to pigs in treatments A to D from day 57 onwards due to the occurrence of scouring in more than 10% of the pigs in each pen.

As this trial was conducted in a commercial herd, normal health management practices were employed to control other diseases. All pigs were vaccinated against *Actinobacillus pleuropneumoniae* (APP) with an autogenous serovar 15 bacterin at 8, 10, 12, and 16 weeks of age. Pigs were fed a commercial grower diet from 9 to 16 weeks of age, followed by a commercial finisher diet from 16 to 22 weeks of age. All feed contained the organic acid Fysal @ 2 kg/t (Selko Feed Additives, Amersfoort, The Netherlands) for Salmonella control and the antimicrobial salinomycin at 60 ppm (BioCox 120 g/kg, Huvepharma, Sofia, Bulgaria) for control of *Brachyspira* spp. Male pigs were vaccinated twice with Improvac (Zoetis, Parsippany, NJ, USA) at 13 and 19 weeks of age. All trial pigs were selected from dams vaccinated against porcine circovirus (PCV2), *Pasteurella multocida*, APP, Erysipelas, Parvovirus, Leptospirosis, Glassers disease, and *Mycoplasma hyopneumoniae*.

### 2.2. Production Measures and Analysis

Pigs were weighed, and their feed intake was measured at six time periods (day 0 on entry, day 21, 42, 56, 70, and 91). The pen weights and number of pigs per pen were recorded and the average daily gain (ADG) was calculated by subtracting the earlier pen weight from the later pen weight and dividing by the number of pigs and number of days between the first and second weights. Average daily feed intake (ADFI) was calculated by subtracting the weight of feed not consumed from the total weight of feed provided over each period and dividing this amount by the number of pigs in the pen and the number of days. The feed conversion ratio (FCR) was calculated as the feed intake divided by the ADG. At slaughter (day 91), the liveweight, hot standard carcass weight (HSCW), the P2 backfat and dressing percent were recorded for each pig. The number of intestines condemned at slaughter was only recorded for the fifth and sixth replicates and expressed as a percent of the total pigs. 

### 2.3. Fecal Sampling, Nucleic Acid Extraction, and Quantitative Polymerase Chain Reaction (qPCR)

Two pooled fecal samples (each containing 5 individual samples randomly selected from the pen floor) were collected from each pen at six time points (day 21, 35, 49, 56, 70, 84). Previous studies demonstrated that random sampling of five fecal samples per pen floor provided an accurate measure of *L. intracellularis* numbers [36]. Nucleic acids were extracted from the feces using a MagMax DNA extraction kit (Applied Biosystems, Foster City, CA, USA) as previously described [37]. Nucleic acids from fecal extracts were amplified in a real-time polymerase chain reaction (RT PCR), alongside fecal standards seeded with known numbers of *L. intracellularis* (10^4^ to 10^8^ *L. intracellularis* per gram of feces). A standard curve from the seeded feces was used to quantify *L. intracellularis* numbers in the pooled pen samples. 

### 2.4. Serum IgG Response to L. intracellularis

Blood from about 11% of the pigs in each pen were collected at three time points (day 30, 63, and 85) to confirm the timing of seroconversion. Serum IgG antibodies to *L. intracellularis* were detected with a commercial competitive (blocking) ELISA kit (Bioscreen^®^ Ileitis, GmbH, Münster, Germany) with a positive index cut-off at 30% inhibition as recommended by the manufacturer.

### 2.5. Statistical Analysis

The effect of treatment on production and disease measures was investigated using an unbalanced analysis of variance (ANOVA) with replicate, gender, and their interactions as blocking effects and starting weight (day 0 = 9 weeks age) as a covariate. The least significance difference (LSD) t test was used post hoc on ANOVA to determine significant differences between treatments, and the average standard error of difference was noted for each analysis. The same data were also modelled with a restricted maximum likelihood linear mixed model (REML) with fixed effects of treatment, gender, starting weight at day 0, and all interactions. Random effects included replicate and interactions between replicate, treatment, gender, and starting weight. Variance for all interactions was constrained to be positive. Significant differences between means were illustrated in figures using the glmmTMB function [38] in the R statistical software (R Core Team 2024, R-4.3.3). A compact letter display (CLD) for pairwise comparisons between treatments for each sex (at each timepoint) was determined using the cld function in the multcomp package [39] with the default Tukey *p*-value adjustment, applied on the marginal means for treatments (within each sex and timepoint) calculated using the emmeans package [40].

Samples below the detection limit for the *L. intracellularis* qPCR were randomly allocated a number under 1000, the limit of quantification of the qPCR. The number of *L. intracellularis* was log_10_ transformed to normalize the distribution of data. A Spearman’s rank correlation (non-parametric) was used to test the association between *L. intracellularis* numbers and the ADG, ADFI, and FCR (Genstat 16th edn, VSN International 2013). Prior to correlation analysis, the ADG was divided by the starting weight at week 9, to compensate for differences between the six replicates.

## 3. Results

### 3.1. Epidemiology of PE in Pigs 9 to 12 Weeks of Age (Trial Days 0 to 21)

Pig starting weights at 9 weeks of age (day 0) were not significantly different between genders, but they were significantly different between replicates, with higher weights in replicates 4 to 6, relative to replicates 1 to 3. There was no serological or qPCR evidence of *L. intracellularis* infection in any of the treatments in the first three weeks of the trial (up to day 21). Neither treatment, nor gender, nor replicate had a significant effect on ADG. However, gender, replicate, and starting weight affected the FCR and ADFI in this period. There was some evidence of APP infection in this period, which caused lost production and mortalities that were not significantly different between treatments.

### 3.2. Epidemiology of PE in Pigs 12 to 15 Weeks of Age (Trial Days 21 to 42)

*L. intracellularis* infection was detected in treatments C (lime) and D (control) at day 35, with these treatments excreting significantly more *L. intracellularis* than treatments A (vaccine + lime), E, F, and G (medicated with 12, 25, and 50 ppm olaquindox, respectively) (Table 2). Control pigs (D) also shed more *L. intracellularis* than pigs in treatment B (vaccine alone). Over this period, pigs across all treatments were clinically affected with APP and swine dysentery, with mortalities ranging from 0.2% to 1.8% of pigs. Swine dysentery accounted for 20 of the 67 deaths, and APP caused 35 of 67 deaths. However, mortalities were not significantly different between treatments.

**Figure 2:**
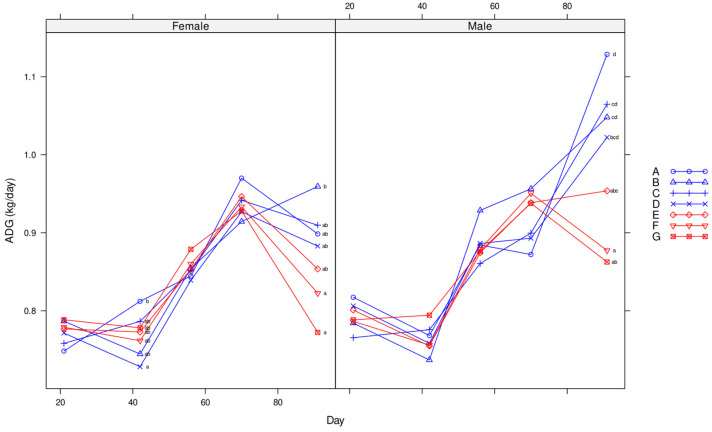
Mean ADG over time for females (**left side**) and males (**right side**) for treatments A to G (Enterisol vaccine plus lime; vaccine only; lime only; no treatment; 12 ppm; 25 ppm; and 50 ppm olaquindox, respectively). Different superscripts indicate significant differences.

ADG was affected by replicate between days 21 and 42, but this may have been a consequence of differences in starting weights, with replicates 4 to 6 heavier than replicates 1 to 3. Although there was no overall treatment effect on ADG, least square differences from the ANOVA indicate that ADG was lower in treatments B (vaccine only) and D (controls) relative to treatments A (vaccine plus lime), C (lime), and G (50 ppm olaquindox) between days 21 and 42. Vaccination alone (B) appeared to have no effect on the ADG, relative to the control, i.e., the untreated pigs. In contrast, vaccination plus lime (A), lime alone (C), and olaquindox treatments (E, F, and G), improved the ADG relative to the control (D) treatment (Figure 2).

Between day 21 and 42, treatment had a significant effect on the ADFI, along with replicate, gender, starting weights, and the interaction between replicate and gender (*p* = 0.05). Vaccinated pigs in lime-treated pens (A) had a higher ADFI than all other treatments (B to F), except treatment G (50 ppm olaquindox). Treatment G pigs had a higher ADFI than the control (D) and vaccinated (B) pigs. Females had a significantly higher ADFI than males (1.69 and 1.59 kg, respectively, *p* < 0.001). Feed intake was also higher in replicates 4 to 6 relative to replicates 1 to 3, which may be explained by higher starting weights in replicates 4 to 6. Feed efficiency was not significantly different between treatments, but both gender and replicate affected the FCR. No significant correlation was observed between the number of *L. intracellularis* excreted (day 21 and 35) and the ADG (R = −0.067, *p* = 0.545), ADFI (R = −0.030, *p* = 0.786), and FCR (R = 0.039, *p* = 0.727) between days 21 and 42. 

### 3.3. Epidemiology of PE in Pigs 15 to 17 Weeks of Age (Trial Days 42 to 56)

**Figure 3:**
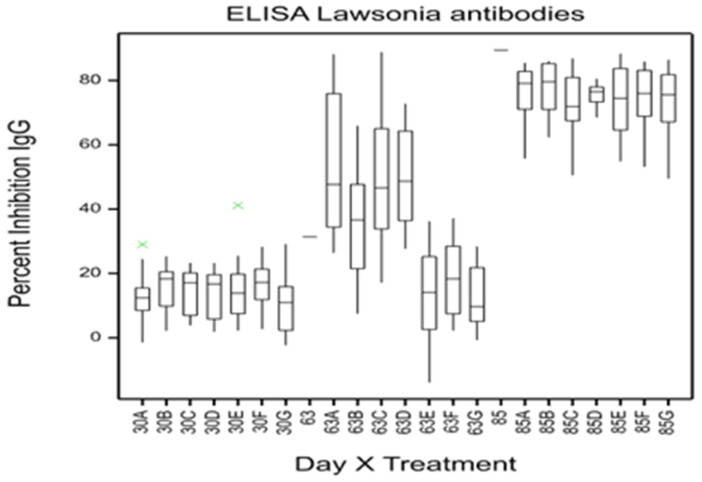
Box and whisker plot of mean serum concentration of IgG antibodies to *L. intracellularis* at day 30, 63, and 85 for treatments A to G (vaccine plus lime; vaccine only; lime only; no treatment; 12 ppm; 25 ppm; and 50 ppm olaquindox). A percent inhibition less than 30% was considered negative. Descriptive statistics were not tested for significance. Green cross indicates outlier value.

Pigs medicated with olaquindox continued to show no evidence of *L. intracellularis* infection until day 56, when *L. intracellularis* were first detected in treatment E (12 ppm olaquindox) feces. Treatments A to D demonstrated a significantly higher excretion of *L. intracellularis* at day 56 and antibodies to *L. intracellularis* at day 63 relative to treatments E, F, and G (medicated with olaquindox) (Table 2 and Figure 3). 

Neither the treatment nor starting weight affected the ADG, ADFI, or FCR. However, there was a gender effect on all production measures (Figure 2), and there was a replicate effect on the ADFI and FCR. Although some diarrhea was observed in treatments A to D between days 42 and 56, coinciding with a significantly higher fecal excretion of *L. intracellularis* (Table 2), no significant differences in the ADG, ADI, or FCR were observed in treatments A to D relative to the olaquindox treatments E, F, and G. There was a significant negative correlation between the number of excreted *L. intracellularis* and the ADG (relative to the starting weight) between days 21 and 56 (R = −0.350, *p* = 0.016). Minimal mortalities occurred in this period.

### 3.4. Epidemiology of PE in Pigs 17 to 19 Weeks of Age (Trial Days 56 to 70)

Diarrhea was observed in treatments A to D around 56 days, and these treatments were medicated in feed with tylan from day 57 onwards. At day 70, significantly higher numbers of *L. intracellularis* were detected in treatments A, B, and C relative to both treatments F and G (*p* = 0.004) (Table 3). *L. intracellularis* numbers in treatments D (control) and E (12 ppm olaquindox) were also higher than treatments F and G (25 and 50 ppm olaquindox) (Table 3). However, neither treatment, gender, nor replicate had a significant effect on the ADG, ADFI, or FCR (Figure 2), but the starting weight did significantly affect the ADFI (*p* < 0.05). There was a significant negative correlation between *L. intracellularis* numbers and the relative ADG between days 56 and 70 (r = −0.580, *p* < 0.001). Minimal mortalities occurred in this period.

### 3.5. Epidemiology of PE in Pigs 19 to 21 Weeks of Age (Trial Days 70 to 84)

Bloody diarrhea was observed from day 79 onwards in treatments E, F, and G, which required treatment of individual pigs with injectable Lincomycin (Lincomix RTU, Zoetis, Kalamazoo, MI, USA). Mortalities due to PE and swine dysentery occurred in all treatments, but the highest mortalities occurred in the olaquindox treatments (E and G: 12 and 50 ppm olaquindox), with mortalities of 3.1% and 4.6%, respectively. However, there was no statistically significant difference in mortalities between any treatments over this period (*p* = 0.149) or over the whole trial period (day 0 to 91).

Excretion of *L. intracellularis* continued to be detected in all groups at days 70 and 84. However, at day 84, significantly higher numbers of *L. intracellularis* were detected in treatments E, F, and G (previously medicated with olaquindox) relative to treatments A to D (Table 3). In addition, the control treatment (D) had the lowest mean number of *L. intracellularis*, which was significantly different to treatments B, E, and F (*p* < 0.001). The mean concentration of antibodies to *L. intracellularis* was not significantly different (*p* = 0.941) between treatments at day 85 (Figure 3).

**Figure 4:**
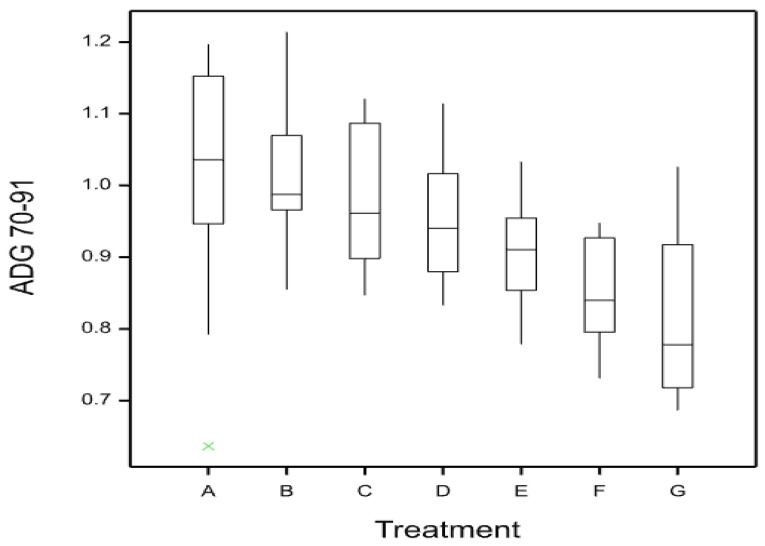
Box and whisker plot of ADG between day 70 and 91 for treatments A to G (Enterisol vaccine plus lime; vaccine only; lime only; no treatment; 12 ppm; 25 ppm; and 50 ppm olaquindox). Descriptive statistics were not tested for significance. Green cross indicates outlier.

Both treatment and gender had a significant effect on the ADG (*p* < 0.001), with a higher ADG for treatments A, B, and C relative to treatments E, F, and G (Figure 4). Higher ADG was also observed in control pigs (D) relative to pigs previously medicated with olaquindox (treatments F and G) (Figure 4). Treatment, gender and replicate all had a significant effect on the ADFI with a lower ADFI in pigs previously medicated with olaquindox (E, F, and G) relative to treatments A and B. The FCR between treatments was close to significantly different (*p* = 0.052) with a numerically higher FCR in pigs previously medicated with 50 ppm olaquindox (G) compared with pigs in treatments B and C. In this period, gender and replicate also had a significant effect on the FCR (*p* < 0.001).

In this final period, there was a strong negative correlation between *L. intracellularis* numbers at day 84 and the ADG (R = −0.573 and *p* < 0.001) and ADFI (R = −0.616 and *p* < 0.001) and a positive correlation with the FCR (R = 0.276 and *p* = 0.011).

### 3.6. Epidemiology of PE in Pigs from 9 to 21 Weeks of Age (Trial Days 0 to 84)

**Figure 5:**
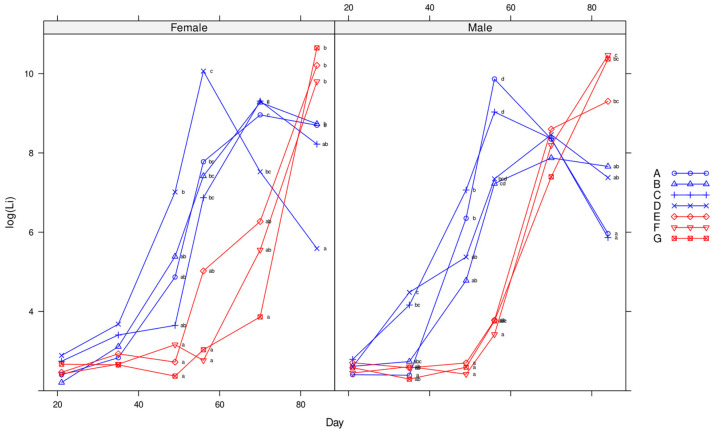
Mean Log_10_ *L. intracellularis* numbers excreted over time for each gender (females on left hand side and males on right side) and treatments A to G (Enterisol vaccine plus lime; vaccine only; lime only; no treatment; 12 ppm; 25 ppm; and 50 ppm olaquindox). Different superscripts indicate significant differences.

Over the whole trial period, the ADG was significantly different between treatments (*p* < 0.001) (Table 4) and between gender (males = 0.8671 and females = 0.8327, *p* < 0.001) (Figure 5). The starting weight, replicate, and interactions between treatment and gender did not significantly affect the ADG. The ADFI was significantly different between treatments (*p* = 0.003), with the highest feed intake in the vaccinated plus lime treatment A and the lowest ADFI in the 25 ppm olaquindox treatment (Table 4). The replicate, gender, and starting weight also had a significant effect on the ADFI, with a higher intake in males relative to females (1.945 and 1.912, respectively). The FCR was not significantly different between treatments over the whole period (*p* = 0.154), but the replicate, gender, starting weight, and treatment–gender interactions did significantly impact the FCR (*p* < 0.001). Males showed a lower FCR than females (2.204 versus 2.337).

The pig liveweight at 91 days and the mean P2 backfat was affected by treatment (*p* = 0.012 and 0.015, respectively) with lower P2 values and lower liveweights in pigs medicated with higher concentrations of olaquindox (Table 5). Carcass measures, including hot standard carcass weight (HSCW) and mean dressing percent, were not affected by treatment (*p* > 0.05). The percentage of intestines condemned at abattoir due to damage from *L. intracellularis* infection was between 0% and 4.48% but was only recorded for the 783 pigs slaughtered in replicates 5 and 6, so the results were not analyzed for significance. The carcass dressing percent, P2 backfat, HSCW, and mean liveweight at 91 days were all affected by the replicate (*p* < 0.012), and all measures except HSCW were also affected by gender (*p* < 0.044). 

## 4. Discussion

Molecular and serological epidemiology tools were able to demonstrate when pens of commercial pigs became infected with *L. intracellularis,* the severity of PE lesions by inference from the numbers of excreted *L. intracellularis*, and the proportion and timing of pigs that raised an immune response to infection. Clinical signs correlated in time with peaks in *L. intracellularis* excretion as previously demonstrated [31], and significant negative correlations were observed between the ADG and excretion of *L. intracellularis* in the periods 21 to 56 days, 56 to 70 days, and 70 to 84 days. Similar negative correlations between the ADG and number of excreted *L. intracellularis* have been reported in fecal samples from individual pigs [33], but this is the first report of this correlation for pens of pigs on commercial farms. Ultimately, these epidemiological tools enabled the comparison of treatment options for the control of PE in a large commercial pig herd.

Medicating all weaner pigs with olaquindox from 3 to 9 weeks of age prevented *L. intracellularis* infection in all pigs prior to the start of the trial, as demonstrated by the absence of *L. intracellularis*-specific antibodies 30 days after treatments commenced. Serum IgG antibodies are routinely detected 14 to 28 days post exposure, depending on the oral dose of *L. intracellularis* [4,41]. As a consequence, this study evaluated PE control treatments in pens of commercially reared pigs between 9 and 22 weeks of age. Pigs in this study were naturally exposed to a relatively low number of *L. intracellularis* after 9 weeks of age, as demonstrated by the low level of *L. intracellularis* excretion in untreated control pigs at 14 weeks of age and the absence of significant production losses in this group between 9 and 12 weeks of age. Clinical PE and the excretion of higher numbers of *L. intracellularis* were not demonstrated in control pigs until they reached 15 to 16 weeks of age (6 to 7 weeks after the trial commenced). In experimental *L. intracellularis* challenge trials, clinical signs of PE and production losses are usually evident between 3 and 4 weeks after exposure [4,42]. In this commercial piggery study, medication, vaccination, and disinfection treatments all impacted on the timing and severity of *L. intracellularis* infection and the associated clinical signs of PE.

Most pigs maintained on high levels of in-feed olaquindox remained naïve to *L. intracellularis* until the medication was removed from their diets, as evidenced by the absence of detectable *L. intracellularis* antibodies at day 63 and low fecal excretion of *L. intracellularis* at days 35, 49, and 56. This agrees with experimental challenge studies, where most pigs medicated with 25 or 50 ppm olaquindox failed to excrete *L. intracellularis* in their feces and were, therefore, susceptible to *L. intracellularis* infection once olaquindox was removed from their diets [29]. Once medication was removed from our commercial herd, naïve finisher pigs developed severe clinical signs, including black tarry feces and death due to intestinal hemorrhage, and they excreted extremely high numbers of *L. intracellularis* (10^10^ per gram of feces compared with the next highest of 10^8.8^). Net revenue is significantly impacted by the death of finisher pigs, where the cumulative costs of feeding pigs from weaning to finishing cannot be recovered. In addition, pigs affected with PHE at 19 to 21 weeks of age did not have sufficient time to recover weight losses before slaughter, and up to 4.5% of their intestines could not be sold for sausage casing. Outbreaks of severe PHE are reported to be associated with the increasing age of infected pigs, rather than any difference in the challenge dose of *L. intracellularis* [6,7]. A range of other antibiotics at high concentrations have been reported to prevent *L. intracellularis* infection, including tiamulin [24], chlortetracycline and oxytetracycline [43], tylosin phosphate [25], and tilmicosin [44]. Field and experimental challenge studies have demonstrated that *L. intracellularis* infection of naïve finisher pigs after the removal of protective antibiotics can cause PHE outbreaks [7,45].

Interestingly, pigs medicated with 12 ppm olaquindox started to excrete higher numbers of *L. intracellularis* at an earlier time than pigs medicated with higher concentrations of olaquindox, suggesting that 12 ppm was not as effective in preventing *L. intracellularis* infection compared with higher levels. As a consequence, pigs medicated with 12 ppm olaquindox were protected from severe clinical signs of hemorrhagic enteropathy and production losses. The ADG between 9 and 22 weeks of age in the pigs medicated with 12 ppm olaquindox was not significantly different to control pigs, vaccinated pigs, and pigs housed in lime-treated pens. 

Neither vaccination alone nor disinfection alone significantly increased the ADG or ADFI nor decreased the feed to gain relative to control pigs over the grower and finisher phases of production, contrary to previous vaccination studies in experimental challenge trials [20]. However, housing vaccinated pigs in lime-treated pens did significantly improve production measures relative to control pigs. The vaccination of pigs in washed or washed and disinfected pens appeared to delay or suppress *L. intracellularis* infection, observed as significantly fewer *L. intracellularis* excreted at day 35 compared with control pigs. However, by days 49 and 56, there were no significant differences in the number of *L. intracellularis* excreted, and by day 70, vaccinated pigs shed significantly more *L. intracellularis* than control pigs. In contrast, vaccination was able to reduce the fecal excretion of *L. intracellularis* in experimental studies where pigs were challenged with *L. intracellularis* at a single time point three weeks after vaccination [20,41]. Under commercial production conditions, pigs may be challenged with *L. intracellularis* many times, and pigs in this trial were vaccinated at least 6 weeks before they were exposed to *L. intracellularis*. Both vaccination and lime disinfection only appeared to suppress *L. intracellularis* infection for a short period around the time of exposure. Challenging pigs with a lower dose of *L. intracellularis* can also suppress *L. intracellularis* infection and clinical disease [3,5,7]. This study suggests that once *L. intracellularis* enter the pigs’ intestinal epithelium and start replicating, antibiotics that can accumulate intracellularly are best at suppressing infection and disease [45]. 

*L. intracellularis* is transmitted between pigs by the fecal-oral route [16,17], so poor pen hygiene and the accumulation of manure increase the dose of *L. intracellularis* that pigs are exposed to. In addition, *L. intracellularis* can survive in feces for at least two weeks and colonize naïve weaner pigs dosed with contaminated feces [16,35]. On-farm epidemiology studies demonstrated that sheds with poor hygiene due to high stocking densities, a continuous flow production, and partially slatted floors increased the risk of *L. intracellularis* infection, while all-in-all production and the use of disinfectants between batches of pigs appeared to protect pigs [13,18,19,46]. Disinfectants like Virkon S can successfully eliminate *L. intracellularis* from contaminated grower pens and can prevent transmission of *L. intracellularis* to naïve pigs in disinfected pens [35]. However, lime disinfection of commercial grower pens failed to significantly reduce *L. intracellularis* excretion over the following 13 weeks relative to other treatments in this study. Housing pigs in lime-disinfected pens also failed to improve pig production measures associated with PE relative to control pigs, which were repeatedly exposed to *L. intracellularis*. 

In contrast, the disinfection of pens in combination with vaccination increased the protection of pigs against PE. Combining the disinfection of commercial pens with antibiotic medication and the partial depopulation of herds has also proved successful in reducing the survival and transmission of *L. intracellularis* in attempts to eradicate PE from small commercial herds [47,48,49]. 

Under commercial conditions, where pigs were housed in washed but not disinfected pens throughout the grower and finisher periods, antibiotic pulses were also required to control clinical signs of PE. Once diarrhea was observed, pigs in all treatments were medicated in feed with tylosin two days of every 10 days from day 56 to 84. Previous studies demonstrate that tylosin can suppress clinical signs of PE and reduce the fecal excretion of *L. intracellularis* and the associated production losses [25,29]. 

For molecular and serological monitoring of commercial herds to be economical, some thought needs to be given to the optimal timing for sample collection. The target sampling period should only commence when preventative antibiotics are removed from diets and before clinical signs of PE are evident. Sampling should continue to the end of the finisher phase. As serological monitoring aims to demonstrate both the timing of the *L. intracellularis* challenge and the proportion of pigs with immunity, blood needs to be collected from about 10% of pigs every three to four weeks. Published *L. intracellularis* serological surveys support this frequency of sampling to identify when pigs are exposed to *L. intracellularis* and to determine the seroprevalence of herds [10]. Pooled pen fecal samples probably need to be collected every two to three weeks, as experimental challenge studies indicate that the fecal excretion of *L. intracellularis* persists for about four weeks, depending on the oral challenge dose [3,4]. In this study, pooled pen fecal samples were collected from every pen every two weeks because the impact of seven different treatments was being investigated over the same period. It may have been possible to reduce the number of sampled pens per treatment in those treatments with 14 pens. This sampling protocol provided sufficient statistical power to demonstrate the efficacy of PE control measures on a commercial herd with 8 to 14 pens per treatments, given the experimental design outlined.

Evaluating PE control on commercial farms can be confounded by the presence of other diseases in the herd. In this study, outbreaks of pleuropneumonia were observed in pigs between 9 and 15 weeks of age, and swine dysentery and PE outbreaks were observed between 15 and 22 weeks of age. As olaquindox is also effective in controlling swine dysentery [50], some of the production gains in olaquindox-treated pigs between 9 and 17 weeks of age may have been due to better control of swine dysentery as well as PE. However, studies have also reported the recurrence of swine dysentery once olaquindox is removed [50], as observed in treatments E, F, and G. 

## 5. Conclusions

Molecular and serological epidemiology tools have been successfully used to identify when pigs in commercial herds are exposed to *L. intracellularis,* the severity of PE they experience, when the majority of pigs have developed immunity, and when treatments are required. Vaccination and the cleaning and disinfection of pens before pigs enter grower facilities can reduce exposure to *L. intracellularis* and can reduce clinical signs of PE and the associated production losses, but PE control may also require antibiotic medication if clinical signs persist. Molecular and serological epidemiology tools can identify the optimal timing for a wide range of treatments. As the pig industry aims to identify management practices that reduce its reliance on antibiotics to control bacterial diseases, this trial has demonstrated a method to evaluate PE control on the farm and also demonstrated that PE can be controlled by a combination of good hygiene and vaccination, with the judicious use of antibiotics if required.

## Figures and Tables

**Table 1:** Treatments and number of pens per treatment.

Treatment Group	Number of Pens	Treatment Description
A	14	Lime disinfectant + vaccination
B	14	Vaccination
C	12	Lime disinfectant
D	8	No treatment
E	14	12 ppm Olaquindox
F	14	25 ppm Olaquindox
G	8	50 ppm Olaquindox
Total	84	

**Table 2:** Mean number of *L. intracellularis* excreted (Log_10_/g feces) at days 35, 49, and 56 for treatments A to G (vaccine plus lime; vaccine only; lime only; no treatment; 12 ppm; 25 ppm; and 50 ppm olaquindox), average standard error of difference (Av SED) and F probability.

Treatment		Log_10_ *L. intracellularis*/g Feces		
Group	Description	Day 35	Day 49	Day 56
A	Disinfectant + vaccination	2.631 ^a^	5.665 ^b^	8.852 ^b^
B	Vaccination	2.925 ^a^	5.111 ^b^	7.351 ^b^
C	Disinfectant	3.741 ^b^	5.136 ^b^	7.783 ^b^
D	No treatment	4.091 ^b^	6.288 ^b^	8.884 ^b^
E	12 ppm Olaquindox	2.753 ^a^	2.702 ^a^	4.372 ^a^
F	25 ppm Olaquindox	2.600 ^a^	2.713 ^a^	3.068 ^a^
G	50 ppm Olaquindox	2.482 ^a^	2.453 ^a^	3.349 ^a^
Av SED		0.4482	0.8706	0.9097
ANOVA		*p* = 0.005	*p* < 0.001	*p* < 0.001

Different superscripts within columns indicate significant differences.

**Table 3:** Mean number of *L. intracellularis* excreted (Log_10_/g feces) at days 70 and 84 for treatments A to G (vaccine plus lime; vaccine only; lime only; no treatment; 12 ppm; 25 ppm; and 50 ppm olaquindox), average standard error of difference (Av SED) and F probability.

Treatment		Log_10_ *L. intracellularis*/g Feces	
Group	Description	Day 70	Day 84
A	Disinfectant + vaccination	8.643 ^c^	7.314 ^bc^
B	Vaccination	8.490 ^c^	8.177 ^b^
C	Disinfectant	8.837 ^c^	7.210b ^c^
D	No treatment	8.191 ^b^	6.390 ^c^
E	12 ppm Olaquindox	7.416 ^b^	9.777 ^a^
F	25 ppm Olaquindox	6.805 ^a^	10.201 ^a^
G	50 ppm Olaquindox	5.723 ^a^	10.555 ^a^
Av SED		0.8081	0.7804
ANOVA		*p* = 0.004	*p* < 0.001

Different superscripts within columns indicate significant differences.

**Table 4:** Predicted ADG, ADFI, and feed conversion efficiency (between day 0 and 91) blocked by replicate and gender with starting weight as a covariate (ANOVA), with standard error of difference (Av SED).

Treatment Group	Treatment Description	ADG	ADFI	FCR
A	Disinfectant + vaccination	0.8714 ^a^	1.990 ^a^	2.287
B	Vaccination	0.8640 ^ab^	1.941 ^ac^	2.248
C	Disinfectant	0.8574 ^ab^	1.934 ^bc^	2.256
D	No treatment	0.8453 ^bc^	1.926 ^bc^	2.280
E	12 ppm Olaquindox	0.8436 ^bc^	1.938 ^abc^	2.298
F	25 ppm Olaquindox	0.8280 ^c^	1.888 ^b^	2.281
G	50 ppm Olaquindox	0.8312 ^c^	1.894 ^bc^	2.280
Av SED		0.01033	0.02602	0.02216

Different superscripts within columns indicate significant differences.

**Table 5:** Predicted mean liveweight, mean backfat, and percent condemned intestines at slaughter (day 91) blocked by replicate and gender with starting weight as a covariate (ANOVA), with average standard error of difference (Av SED).

Treatment Group	Treatment Description	Mean Liveweight 91 Days (kg)	Mean P2 Backfat (mm)	% Condemned Intestines *
A	Disinfectant + vaccination	102.3 ^a^	12.34 ^a^	2.29
B	Vaccination	101.5 ^ab^	12.14 ^ad^	0.85
C	Disinfectant	100.6 ^abd^	12.41 ^a^	1.56
D	No treatment	99.9 ^bc^	11.71 ^bd^	0
E	12 ppm Olaquindox	100.0 ^bc^	11.88 ^abd^	1.96
F	25 ppm Olaquindox	98.7 ^cd^	11.49 ^bc^	2.53
G	50 ppm Olaquindox	98.0 ^c^	11.89 ^abd^	4.48
Av SED		1.165	0.3066	

Different superscripts within columns indicate significant differences. * % intestines condemned due to intestinal hemorrhage only scored for replicates 5 and 6.

## Data Availability

Data is unavailable due to privacy restrictions.
